# 

**Published:** 1971-04

**Authors:** 


The
Bristol Medico-Chirurgical Journal
(Incorporating the Medical Journal of the South-West)
Established 1883

Vol. 86 (ii) APRIL 1971 No. 318

### BRISTOL MEDICO-CHIRURGICAL JOURNAL

Editor: Dr. N. J. Brown, M.B., F.R.C.P., F.R.C.Path.

Published Quarterly

Annual Subscription 80p (16/-) Post Free

### BRISTOL MEDICO-CHIRURGICAL SOCIETY

**President 1970-71 : Dr. Beryl Corner**

**President-elect : Dr. C. C. Morgans**

**Honorary Secretary : Dr. I. S. Bailey, 7 Percival Road, Bristol 8.**

**Honorary Treasurer : Dr. Frank Ross, Bristol Royal Infirmary, Bristol 2.**

The Society was founded in 1874. In 1883 publication of the Journal began and has continued quarterly ever since then. During the years 1949 to 1962 it appeared under the title of "The Medical Journal of the South West".

### THE BRISTOL MEDICO-CHIRURGICAL JOURNAL

### Editorial Committee

| | |
|---|---|
| Editor | Dr. N. J. Brown, Southmead Hospital, Bristol. |
| Assistant Editor | Mr. J. B. M. Roberts. |
| Members | Dr. M. B. Lennard. |
| | Professor R. C. Wofinden. |
| | Dr. D. W. Wright. |
| | The Hon. Treasurer and Hon. Secretary of the Society |
| Business Manager | Dr. P. J. F. Baskett, Frenchay Hospital, Bristol. |
| Secretary | Mr. B. P. Jones, The Library, Medical School, University Walk, Bristol BS8 1TD. |

### NOTICE TO CONTRIBUTORS

The Journal is especially concerned to provide a place for the recording of medical thought, interests, and practice in and around Bristol. It therefore welcomes articles that, as well as being of immediate interest to members, will document the local and contemporary medical scene for our successors.

Original articles are invited provided that they have not been published elsewhere. References in the text should be by author's name, with year of publication in parentheses; they should be listed at the end in alphabetical order, the name of each journal or book being given in full — not abbreviated — and the title of the article added. Illustrations should be supplied ready for reproduction. Line drawings should be in Indian ink on firm white paper or board. The author will be informed if it is found necessary to ask him to pay for the making of blocks.

The following contributions will be especially welcome: notes of forthcoming events, meetings held, degrees conferred, staff appointments, honours and public appointments and other local news.

Twenty-five reprints of his article will be supplied free to each contributor. Quotations for larger quantities will be sent out with proofs and must be ordered when the corrected proofs are returned.

### Bristol Medico-Chirurgical Society

The fourth meeting of the session was held on 13th January, 1971. Dr. J. Briggs showed pathological specimens and Dr. J. L. G. Thomson demonstrated skiagrams. Dr. Glin Bennet, Lecturer in Mental Health and Mr. R. W. Pigott, Consultant Plastic Surgeon, spoke on "Plastic Surgery : Mind over Matter".

At the fifth meeting on 10th February, Mr. C. A. Brown read a paper on "Glaucoma". Pathological specimens were shown by Dr. D. H. Johnson and skiagrams by Dr. J. B. Penry and Mr. V. J. Marmion. Mr. Marmion and Mr. J. C. Dean Hart also showed a demonstration of Fluorescein Angiography of the Optic Fundus.

At both meetings Mr. B. P. Jones arranged his usual interesting display of medical books.

### Wessex Branch of the Association of Clinical Pathologists

A meeting was held at Southmead Hospital, Bristol, on 7th November, 1970. The following papers were read :

Dr. J. Verrier-Jones and Dr. B. Moore — Leuco-allo-antigens in the kidney.

Dr. T. Hamblin — Erythroleukaemia.

Dr. I. D. Fraser — Antibody Quantitation in Rh. Haemolytic Disease.

Dr. O. A. N. Husain (guest speaker) — An Automated Cytology System.

The afternoon was devoted to demonstrations arranged by the medical, scientific and technical staff of the hospital.

### The Medical Library

In 1890 the Bristol Medico-Chirurgical Society founded a medical library which in 1892 was combined with that of the Bristol Medical School. This became the University of Bristol Medical Library in 1925 and an agreement in that year confirmed the privilege of Members of the Society to use the University Medical Library.

### A Selection of Recent Additions to the Medical Library

BALLANTYNE, J. C.: Deafness. Ed. 2. (Churchill)

BEECHER, H. K.: Research and the individual. (Churchill).

BENARDE, M. A. ed.: Disinfection. (Dekker)

BLOOM, S. W.· The doctor and his patient. (Russell Sage Foundation)

BRYAN, G. J.: Diagnostic radiography. (Livingstone)

BURNET, Sir F. M.: Immunological surveillance. (Pergamon)

CALNE, D. B.: Parkinsonism: physiology, pharmacology and treatment. (Ed. Arnold)

CERASI, E. and LUFT, R. eds.: Pathogenesis of diabetes mellitus. (Interscience)

CIBA FOUNDATION: Alzheimer's disease and related conditions. (Churchill)

CIBA FOUNDATION: Breathing: Hering-Breuer centenary symposium. (Churchill)

COHEN, A.: Textbook of medical virology. (Blackwell)

COHEN, S.: Drugs of hallucination. (Secker & Warburg)

CONROY, R. T. W. L. and MILLS, J. N.: Human circadian rhythms. (Churchill)

COWELL, C. R. et al.: Inlays, crowns, and bridges. Ed. 2 (Wright). Presented by John Wright & Sons Ltd.

COWIE, V. A.: A study of the early development of mongols. (Pergamon)

CRATTY, B. J. and MARTIN, M. M.: Perceptual-motor efficiency in children. (Lea & Febiger).

DALLY, P.: Anorexia nervosa. (Heinemann)

EASTHAM, R. D.: Clinical haematology. Ed. 3. (Wright). Presented by John Wright & Sons Ltd.

EGDAHL, R. H. and MANNICK, J. A.: Modern surgery. (Grune & Stratton).

EIMERL, T. S. and LAIDLAW, A. J.: A handbook for research in general practice. Ed. 2. (Livingstone)

FREIDSON, E.: Patients' views of medical practice. (Russell Sage Foundation)

GARRAD, J. and ROSENHEIM, M.: Social aspects of clinical medicine. (Baillière)

GEBHARDT, L. P.: Microbiology. Ed. 4. (Mosby)

GOLDMAN, K. P.: The chest in health and disease. (Health Horizon, for Chest and Heart Association)

GOLDMAN, M. and COPE, D.: A radiographic index. Ed. 4. (Wright). Presented by John Wright & Sons Ltd.

GOODMAN, R. M. ed.; Genetic disorders of man. (Little, Brown & Co.)

HALLAS, C. H.: The care and training of the mentally subnormal. Ed. 4. (Wright). Presented by John Wright & Sons Ltd.

HARRIS, E. L. and FITZGERALD, J. D.: Principles and practice of clinical trials. (Livingstone)

HARRIS, H.: The principles of human biochemical genetics. (North-Holland)

HARRIS, R. J. C.: What we know about cancer. (Allen & Unwin). Presented by the Association of Applied Biologists

HARRIS, W. H. and HEANEY, R. P.: Skeletal renewal and metabolic bone disease. (Churchill)

HAXTON, H.: Surgical techniques (Wright). Presented by John Wright & Sons Ltd.

HELLER, H. and PICKERING, B. T. eds.: Pharmacology of the endocrine system and related drugs: the neurohypophysis. (Pergamon)

HEMKER, H. C. et al. eds.: Human blood coagulation. (Leiden Univ. Pr.)

HOWELL, T. H.: A student's guide to geriatrics. Ed. 2. (Staples)

HUGHES, W. H. and STEWART, H. C.: Concise antibiotic treatment. (Butterworth)

IRVINE, W. J.: Reproductive endocrinology. (Livingstone)

JENNETT, W. B.: Introduction to neurosurgery. Ed. 2. (Heinemann)

JULIAN, D. G.: Cardiology. (Baillière). Presented by Professor C. Bruce Perry

KILBOURNE, E. D. and SMILLIE, W. G.: Human ecology and public health. Ed. 4. (Collier-Macmillan)

LANCE, J. W.: A physiological approach to clinical neurology. (Butterworth)

LEMBECK, F. and SEWING, K. F.: Pharmacological facts and figures. (Longmans)

LEWIS, A. E.: Principles of hematology. (Appleton)

LUSTED, L. B.: Introduction to medical decision making. (Thomas)

MAUER, A.: Pediatric hematalogy. (McGraw-Hill)

MAYERS, M. R.: Occupational health. (Livingstone)

MILES, T. R.: On helping the dyslexic child. (Methuen)

MITTLER, P. ed.: The psychological assessment of mental and physical handicaps. (Methuen)

O'CALLAGHAN, S.; Drug addiction in Britain. (Hale)

PACK, G. T. and ISLAMI, A. H. eds.: Tumors of the liver. (Heinemann)

PATON, A.: Liver disease. (Heinemann)

PENFIELD, W. and JASPER, H. H.: Epilepsy and the functional anatomy of the human brain. (Churchill)

PHILIPP, E. E. et al.: Scientific foundations of obstetrics and gynaecology. (Heinemann)

PICKERING, Sir G.: Hypertension. (Churchill)

RICHARDS, R. L.: Peripheral arterial disease. (Livingstone)

SCHIFF, L.: Disease of the liver. Ed. 3. (Blackwell)

STEPTOE, P. C.: Laparoscopy in gynaecology. (Livingstone)

TULLEY, W. J. and CAMPBELL, A. C.: Manual of practical orthodontics. Ed. 3. (Wright). Presented by John Wright & Sons Ltd.

WOODRUFF, A. W.: Alimentary and haematological aspects of tropical diseases. (Arnold)

ZIAI, M. et al. eds.: Pediatrics. (Churchill)

### News And Notes

### APPOINTMENTS

M. S. Knapp, M.D., M.R.C.P., Consultant Physician, Nottingham Hospitals.

D. S. Reeves, M.B., B.S., M.R.C.Path., Consultant Microbiologist, Southmead Hospital, Bristol.

C. R. Tribe, M.A., D.M., M.R.C.Path., Consultant Pathologist (Morbid Anatomy and Histopathology), Southmead Hospital, Bristol.

### EXAMINATION RESULTS AND DEGREES CONFERRED

**UNIVERSITY OF BRISTOL**

**Ph.D.** (Faculty of Medicine)

C. J. F. Davis, Linda I. L. Fawke, E. P. J. Gibbs, Helga E. T. Stanbury.

**M.B., Ch.B.**

N. J. Hodson (with honours), Manju Bhavnani, S. W. Blackwell, J. D. H. Boulnois, Vanda Boyd, J. A. James, Joyce E. B. Madigane, P. J. Morris, G. Mukasa-Kyannamba, A. W. Pantlin, V. J. Phillips, P. W. G. Saunders, Cheryl U. Wood, J. C. Campbell.

